# Supplementary material for: Marine species and assemblage change foreshadowed by their thermal bias over Early Jurassic warming
Source: Nat Commun. 2025 Feb 5;16:1370. doi: 10.1038/s41467-025-56589-0 (PMC11799210; doi:10.1038/s41467-025-56589-0)
Supplement: Supplementary file 1 — Supplementary Information [file 41467_2025_56589_MOESM1_ESM.pdf]

Supplementary Information to "Marine species and assemblage change foreshadowed by their thermal bias over Early Jurassic warming"

Carl J. Reddin et al.

This document provides information that supplements our paper, "Marine species and assemblage change foreshadowed by their thermal bias over Early Jurassic warming". It is structured into Supplementary Notes, Supplementary Methods, Supplementary References, Supplementary Figures, and Supplementary Tables.

Supplementary Note 1: Influence of extinctions and originations

At our temporal resolution, the best interpretations of climate from geochemical indicators (see Supplementary Methods 1) indicated a gradual warming of climate, from a cold Late Pliensbachian into a warming early Toarcian (finer resolution variations are here averaged out). This gradual warming trend may make a relationship between species thermal bias and its occupancy response seem inevitable because the species that were permanently removed have older and thus colder occurrences, at a given latitude, while first appearance species have only future and thus warmer occurrences. However, the relationship remained even when focussing on species that had both past and future occurrences (i.e. that didn't originate or go extinct) to inform their thermal niche estimations (Supplementary Table 1). The relationship between occupancy response and thermal bias was, nevertheless, stronger when originations and extinctions were included as separate responses (Supplementary Table 2).

Supplementary Note 2: Main results under alternative paleogeographic reconstructions and CO_2 scenarios

Under the alternative CO_2 scenario (generally assuming larger CO_2 changes through time) the results are very similar except increasing the response differences between brachiopods and bivalves (Supplementary Table 3). Using the Pliensbachian paleogeography rather than the Toarcian leads to a slightly stronger effect of thermal bias on occupancy response (Supplementary Table 3). Thus, the main text results can be considered conservative.

Supplementary Note 3: Main results using three-timer method

Alternative methodology, i.e. utilising three-timer species rather than two-timer species, shows that species' thermal bias still correlates with an occupancy response gradient over the focal times of Spinatum, Tenuicostatum, and Exaratum (sub)zones, which are all 3-bin net warming intervals, but not over the Falciferum, which is a 3-bin net cooling interval (Supplementary Fig. 1).

Supplementary Note 4: Influence of number of species, facies, and clade on thermal bias – occupancy response relationship

Changes in sea level over long time scales can move suitable habitat for less generalist species in and out of well-sampled outcrops affecting the observed patterns of species occupancy [1]. Although averaging over many outcrops to regional scale will dilute this effect, we explored how changes in habitat sampling might affect some or all of the two-timer species we analyse.

Changes in the representation of collections from deeper waters were concentrated across the Pliensbachian-Toarcian boundary (Spinatum to Tenuicostatum zones; Supplementary Table 5). The regions east and west of Iberia and the British basins switched from mostly shallow to nearly completely deep environments (46% to 90% deep occurrences in east of Iberia; 18 to 96% deep occurrences in west of Iberia; 28 to 94% deep occurrences in British basins). East and west of Iberia stayed nearly completely carbonate throughout while the Germanic and British basins stayed nearly completely siliciclastic throughout. North of Iberia had the largest lithology changes (by ~35% both from Spinatum to Tenuicostatum, and from Tenuicostatum to Exaratum) but stayed mostly carbonate and shallow. Regional-scale sampling probability generally remained high around Iberia but was lower in the British basins in the Toarcian (Supplementary Table 6).

The relationship between thermal bias and occupancy response remained significant within two-timer species that had a significant affinity to substrate type or water depth categories ($P < 0.0001$), and also if occurrences of these species were removed (still $P < 0.0001$). While a direct test of the effect of substrate changes found no significant effect on species' occupancy response, a significant effect of change in the proportion of depth categories was observed (Supplementary Table 4A). The most substantial increase in deep occurrences was over the Spinatum-Tenuicostatum boundary in the British basins and west and east of Iberia (Supplementary Table 5). This 'deepening' event coincided with a large number of immigrations east of Iberia (see Supplementary Fig. 3), which had a low sampling completeness value of 0.5 through the Spinatum zone (Supplementary Table 6). This implies that some of the immigrations may have truly been unsampled persisting species. Nevertheless, the effect of thermal bias remained highly significant if this depth change is accounted for (Supplementary Table 4A), and also if east of Iberia is removed from the analysis (Supplementary Table 4B).

Thermal bias was a significant predictor of species occupancy response for both bivalves and rhynchonelliform brachiopods analysed separately (both $P < 0.0001$). To supplement interpretation of the interactions in Table 1, brachiopods have a steeper slope for thermal bias ($R = -0.13$, $P <$

0.0001) when analysed separately to bivalves (R = -0.07, P < 0.0001). There were too few gastropods or lingulid brachiopods for a separate analysis. Bivalves had 275 observations of a species response per region per warming or transitional phase, while brachiopods had 146 observations.

Supplementary Note 5: Cool- and warm-adapted thermal bias results

We also calculated assemblage thermal bias as distance of environmental temperatures from quantiles other than the median, which shifts the focus to the assemblages' relatively cool- or warm-adapted species, and whether this helps to predict aspects of assemblage turnover (Supplementary Table 7). Although no strong results differences were detected among thermal bias quantiles of assemblages (e.g. only the proportion of originations is significantly correlated for each quantile of thermal bias), focussing on cool-adapted species seemed to make thermal bias a slightly better predictor of the proportion of an assemblage persisting or being extirpated through warming or transition phases.

Supplementary Note 6: Model-based expectations

Fig. 3 shows how each +1 degree of regional temperature change changed the slope of the relationship between thermal bias and species occupancy response (e.g. the slope in Fig. 2), increasing the thermal bias difference between response levels by 0.25 degrees (regression from species threshold = 20). +3°C regional warming gives a mean thermal bias difference of 0.75°C (95% CIs = 1.46—0.02) between response levels, for example between species that will persist or be extirpated. Again, this may be a conservative estimate because observed thermal biases of extirpated species were significantly lower (cooler) than expected. This suggests a difference between persisting and extirpated ranks more like 1.49°C (95% CIs = 2.92—0.05) under warming. These values imply that extirpations will tend to be from cool-adapted species (increasingly so as the region warms).

A warming of +3°C would predict an overall assemblage thermal bias of -1.24°C (95% CIs = -2.32—-0.16°C, given by 3 x -0.41°C from the main text). Using this thermal bias value with Fig. 4 predicts 4.74% (0.03—9.45%) of an assemblage's pre-existing benthic species to be extirpated (the coefficients in Fig. 4 suggest +3.8% of an assemblage is extirpated per ±1°C increment in assemblage thermal bias, 95% CIs = 0.02—7.6%). Again using Fig. 4, +3°C warming would predict 25.5% (95% CIs = 12.5—38.4%) of an assemblage could be newly immigrated.

A regional warming of +3.11°C (ensemble mean with ±0.98°C internal variability given by 2 times the ensemble standard deviation) is to be expected in the North Sea by the end of the century (2079–2098) over conditions from 2000–2019 following the RCP8.5 scenario [4].

Supplementary Note *7:* Salinity variation from HadCM3 model

The HadCM3 model was only run at two CO_2 levels, but CO_2 levels had the smallest effect on salinity, with higher CO_2 assumptions increasing salinity only by between 0.3 and 0.6 ppt (Supplementary Table 8). Location was the largest cause of regional salinity difference, surpassing the effects of paleogeographical assumption (e.g. tectonic configuration) and CO_2 level. East of Iberia always had the highest salinity, ranging between 34 and 35.6 ppt across models, while the Germanic and British basins had slightly lower salinity, ranging between 33.3 and 34.6 ppt, likely representing much higher seasonal variability. Paleogeographical assumptions had the next largest effect, varying salinity by mean 0.83 ppt across regions.

Supplementary Note 8: Proxy – climate model agreement under different CO_2 scenarios

Focussing on occurrence paleocoordinates, the southern-most two regions, east and west of Iberia, were consistently the warmest, with July seawater surface means of 25–26°C at 400 ppm CO_2 (late Pliensbachian 'cold stasis'). The British and Germanic regions experienced the largest warming magnitudes, and also the greatest transitional phase cooling (i.e. T-OAE interval to subsequent time intervals of -2—-3°C). Regional differences remained generally consistent regardless of the paleoconfiguration or CO_2 scenario used (Supplementary Table 9).

Differences between models were greatest at the northern, coastal edge, consistently over both paleogeographic reconstructions and low and high CO_2 levels. This potentially affected some occurrences of the Germanic basins and British basins regions (the latter only in the Pliensbachian; Supplementary Fig. 7). These were expected, where both global models struggled to model fine-scale coastal mechanics. The Root Mean Squared Error (RMSE) increased with differences in CO_2 levels in the two models, although CLIMBER-X maps tended to be warmer for a given CO_2 concentration. RMSE was lowest between CLIMBER-X at 750 ppm and HadCM3 at 950 ppm (high CO_2 levels), and between CLIMBER-X at 400 ppm and HadCM3 at 560 ppm (low CO_2 levels). Paleogeography made

little difference but correlation was highest between maps under low CO_2 levels, with greater uncertainty suggested for the northern coastal waters. When comparing the two models between contrasting scenarios (Supplementary Table 10), correlations were lowest for CLIMBER-X high CO_2 runs, highlighting uncertainties in the northern coastal waters. HadCM3 is a more complex and more highly resolved model [5,6], and in our context may model regional equilibrium climate conditions better (although this also depends on uncertain local features in the paleogeographic reconstructions).

Supplementary Methods 1: Climate conditions of our major time steps

Direct estimates of atmospheric CO_2 based on proxies are available for the early Toarcian [7–10], suggesting 300-500 ppm in the Tenuicostatum Zone, rising to 1200-1700 ppm during the T-OAE. These time intervals became the anchor for subsequent temperature changes, especially the Tenuicostatum Zone. CO_2 levels for other time intervals were therefore experimentally estimated by matching seawater temperature changes between CO_2 forced models and temperature change proxies, especially $\delta^{18}O$ of well-preserved calcitic shells. In particular, Müller et al. [9] and Ullmann et al. [11] present $\delta^{18}O$ curves that together covered the complete temporal extent of this study, respectively at the Peniche section, Portugal, and the Barranco de la Cañada section, Spain (respectively within our regions west of Iberia and east of Iberia). Proxy based temperature and CO_2 estimates at the temporal resolution of biostratigraphic zones / subzones can be summarised as the following steps. A simple numerical summary is precluded by the wealth of contextual factors that need accounting for to understand the proxy estimate (e.g. depth, geography, temporal resolution, confidence).

1. **<u>Margaritatus</u>**

Margaritatus and Spinatum Zone CO_2 estimates were set as equal because, even if Margaritatus and Spinatum had different SST averages, the variation between (and among) them was smaller than the change from the Spinatum to the Tenuicostatum zones (earliest Toarcian). This includes reported late Pliensbachian cooling, likely occurring at the end of the Margaritatus Zone [12].

2. **<u>Spinatum Zone (late Pliensbachian), duration 1.4 ma</u>** [13]

A Spinatum mean estimate for bottom water temperature around 25 degrees latitude is 15.4°C from rhynchonellid brachiopods [12]. Reporting from 5-10 degrees latitude further south, Ruebsam et al. [10] give Emaciatum Zone (for our purposes equal to Spinatum) SST means of ~27°C. They used TEX86, the reliability of which is unclear in the Mesozoic but would correspond to 'summer' sea surface temperature (i.e. usually higher than annual average but here the climate is tropical anyway). Data given in Korte & Hesselbo [14] and Danise et al. [15] suggest a gradual warming in the Spinatum, cautioning that temperatures where not stable, but were nonetheless colder than at the stage boundary.

3. **<u>Tenuicostatum (= Polymorphum) Zone (early Toarcian), duration ca. 900 kyr</u>** [16]

Although too short for our analysis to infer on, we mention the negative $\delta^{13}C$ spike of ~120 kyr duration [17] that marks the Pliensbachian-Toarcian boundary as its permanent effects may impact our results. $\delta^{18}O$ suggests a rapid warming of shallow shelf sea bottom waters from 16.2 to 21.2°C, a warming of 5.1 degrees [12], or alternatively and at slightly lower latitudes, TEX86 suggest SST warming from 25 to 30°C [10].

The rhynchonellid brachiopod data from Suan et al. [12] suggest an increase of 2.5°C from the Spinatum mean to a Polymorphum bottom water mean of ~24°C (bottom water temperatures for the same basin were cooler at ~21°C in Ullmann et al. [11]). Our models suggest this could indicate a doubling of CO_2 to around the ~600 ppm reported by McElwain et al. [7], based on conifer and gingko stomatal index. We expect the Tenuicostatum Zone to have perhaps the most stable conditions of the overall time interval (Ullmann pers comms).

4. Exaratum (= Elegantulum) Subzone: 790 – 1012 / 1080 kyr [18]

The T-OAE (early Serpentinum Zone) is estimated a carbon isotope excursion duration of 400 – 500 kyr (see e.g. Remirez and Algeo [19] for summary of estimates). Based on arguably the best and most continuous brachiopod data for the early Toarcian, Ullmann et al. [11] conclude that *"... oxygen isotope data from calcite shells of the benthic fauna suggest that [shallow shelf] bottom water temperatures in the western Tethys were elevated by c. 3.5 °C through the entire T-OAE*", suggesting a doubling of CO_2 levels. McElwain et al. [7] suggest a pCO_2 of ~1350 ppm at the peak of the T-OAE (ignoring the short CO_2 draw-down and cooling event just prior), accompanied by a global mean land surface temperature rise of 4.5 °C. These suggest our model settings at 1000ppm may even be conservative for the Exaratum. Based on TEX86, Ruebsam et al. [10] report the T-OAE onset to show a 10 °C rise (from 22 to 32 °C) in tropical SST, which is more than our models allow for. However, many of the temperature peak estimates are at a finer temporal resolution than the zone resolution of our analysis.

Foster et al. [20] review evidence that supports the T-OAE having an onset duration of ∼150 kyr, a total duration of warmth/$\delta^{13}C$ excursion of ~300 kyr, and low-latitude SST warming of 2-5 degrees. The modelling experiment study of Dera & Donnadieu [21] concluded that, *"by assuming a tripling of* p*CO_2 levels during the Early Toarcian, the average global air temperature increases by +4.5°C, ranging from 15.5°C at 2× CO_2 to 20°C at 6× CO_2".*

5. Falciferum

The total duration of the Serpentinum (= Levisoni) Zone is estimated at 2 myr (Suan et al. [16]; min. 1500 – 1620 kyr, Boulila & Hinnov [18]), which includes the Exaratum and Falciferum subzones. After the T-OAE, Ullman et al. [11] then estimate bottom water temperatures cooling by around 1.6°C for the

remainder of the Serpentinum Zone, thus remaining around 2°C higher than during the Polymorphum Zone.

6. **Bifrons**

Most publications show little temperature change from the Falciferum into the Bifrons Zone, but do not cover the whole Bifrons Zone [11,12,22].

Supplementary Methods 2: Climate model (dis)agreement

Although the broad temperature trends from a global climate model are likely to be robust, marine regions with restricted flow, nutrient traps, stratification, and other factors are likely to increase system complexity and decrease model accuracy due to limited spatial resolution [23,24]. Therefore, temperature estimates of the British and Germanic basins, lying at the mouth of the Viking seaway between the Boreal Ocean and the Tethys, may be less accurate than more open-ocean influenced regions. Although the low availability of fossils during some phases limited inference here anyway, these northern regions are also covered by the regional circulation model of Bjerrum et al. [25], which we used to supplement our understanding of regional conditions. Otherwise, Judd et al. [23] caution that temperatures in epeiric seas tend to be warmer and more seasonal in reality than the modelled zonal means, while their biochemical proxies may also be seasonally biased. Even the relatively well-resolved BRIDGE suite of models can develop local errors of over 8°C where local processes are unresolved by the model, such as eastern boundary upwelling currents and sharp fronts [5]. Modelling additional processes requires higher spatiotemporal resolutions [24] but could lead to more robust quantification of thermal bias.

Supplementary References

1. Patzkowsky, M. E. & Holland, S. M. *Stratigraphic paleobiology : understanding the distribution of fossil taxa in time and space*. (The University of Chicago Press, 2012).

2. Foote, M. & Raup, D. M. Fossil preservation and the stratigraphic ranges of taxa.pdf. *Paleobiology* **22**, 121–140 (1996).

3. Alroy, J. Dynamics of origination and extinction in the marine fossil record. *Proc. Natl. Acad. Sci.* **105**, 11536–11542 (2008).

4. Cornes, R. C. *et al. Climate change impacts on temperature around the UK and Ireland. MCCIP Science Review 2023*. (2023) doi:10.14465/2022.reu08.tem.

5. Valdes, P. J. *et al.* The BRIDGE HadCM3 family of climate models: HadCM3@Bristol v1.0. *Geosci. Model Dev.* **10**, 3715–3743 (2017).

6. Willeit, M., Ganopolski, A., Robinson, A. & Edwards, N. R. The Earth system model CLIMBER-X v1.0 – Part 1: Climate model description and validation. *Geosci. Model Dev.* **15**, 5905–5948 (2022).

7. McElwain, J. C., Wade-Murphy, J. & Hesselbo, S. P. Changes in carbon dioxide during an oceanic anoxic event linked to intrusion into Gondwana coals. *Nature* **435**, 479–482 (2005).

8. Nordt, L., Breecker, D. & White, J. Jurassic greenhouse ice-sheet fluctuations sensitive to atmospheric CO2 dynamics. *Nat. Geosci.* **15**, 54–59 (2022).

9. Müller, T. *et al.* Ocean acidification during the early Toarcian extinction event: Evidence from boron isotopes in brachiopods. *Geology* **48**, 1184–1188 (2020).

10. Ruebsam, W., Reolid, M., Sabatino, N., Masetti, D. & Schwark, L. Molecular paleothermometry of the early Toarcian climate perturbation. *Glob. Planet. Change* **195**, 103351 (2020).

11. Ullmann, C. V. *et al.* Warm afterglow from the Toarcian Oceanic Anoxic Event drives the success of deep-adapted brachiopods. *Sci. Rep.* **10**, (2020).

12. Suan, G. *et al.* Secular environmental precursors to Early Toarcian (Jurassic) extreme climate changes. *Earth Planet. Sci. Lett.* **290**, 448–458 (2010).

13. Ruhl, M. *et al.* Astronomical constraints on the duration of the Early Jurassic Pliensbachian Stage and global climatic fluctuations. *Earth Planet. Sci. Lett.* **455**, 149–165 (2016).

14. Korte, C. & Hesselbo, S. P. Shallow marine carbon and oxygen isotope and elemental records indicate icehouse-greenhouse cycles during the Early Jurassic. *Paleoceanography* **26**, (2011).

15. Danise, S. *et al.* Stratigraphic and environmental control on marine benthic community change through the early Toarcian extinction event (Iberian Range, Spain). *Palaeogeogr. Palaeoclimatol. Palaeoecol.* **524**, 183–200 (2019).

16. Suan, G., Van De Schootbrugge, B., Adatte, T., Fiebig, J. & Oschmann, W. Calibrating the magnitude of the Toarcian carbon cycle perturbation. *Paleoceanography* **30**, 495–509 (2015).

17. Boulila, S., Galbrun, B., Sadki, D., Gardin, S. & Bartolini, A. Constraints on the duration of the early Toarcian T-OAE and evidence for carbon-reservoir change from the High Atlas (Morocco). **175**, 113–128 (2019).

18. Boulila, S. & Hinnov, L. A. A review of tempo and scale of the early Jurassic Toarcian OAE: implications for carbon cycle and sea level variations. *Newsletters Stratigr.* **50**, 363–389 (2017).

19. Remírez, M. N. & Algeo, T. J. Carbon-cycle changes during the Toarcian (Early Jurassic) and implications for regional versus global drivers of the Toarcian oceanic anoxic event. *Earth-Science Rev.* **209**, 103283 (2020).

20. Foster, G. L., Hull, P., Lunt, D. J. & Zachos, J. C. Placing our current 'hyperthermal' in the context of rapid climate change in our geological past. *Philos. Trans. A. Math. Phys. Eng. Sci.* **376**, 20170086 (2018).

21. Dera, G. & Donnadieu, Y. Modeling evidences for global warming, Arctic seawater freshening, and sluggish oceanic circulation during the Early Toarcian anoxic event. *Paleoceanography* **27**, (2012).

22. Piazza, V., Ullmann, C. V. & Aberhan, M. Temperature-related body size change of marine benthic macroinvertebrates across the Early Toarcian Anoxic Event. *Sci. Rep.* **10**, 1–13 (2020).

23. Judd, E. J., Bhattacharya, T. & Ivany, L. C. A Dynamical Framework for Interpreting Ancient Sea Surface Temperatures. *Geophys. Res. Lett.* **47**, (2020).

24. Holt, J. *et al.* Modelling the global coastal ocean. *Philos. Trans. R. Soc. A Math. Phys. Eng. Sci.* **367**, 939–951 (2008).

25. Bjerrum, C. J., Surlyk, F., Callomon, J. H. & Slingerland, R. L. Numerical paleoceanographic study of the Early Jurassic Transcontinental Laurasian Seaway. *Paleoceanography* **16**, 390–404 (2001).

26. Foote, M. Origination and extinction components of taxonomic diversity: general problems. *Paleobiology* **26**, 74–102 (2000).

27. Alroy, J. Accurate and precise estimates of origination and extinction rates. *Paleobiology* **40**, 374–397 (2014).

Supplementary Figures

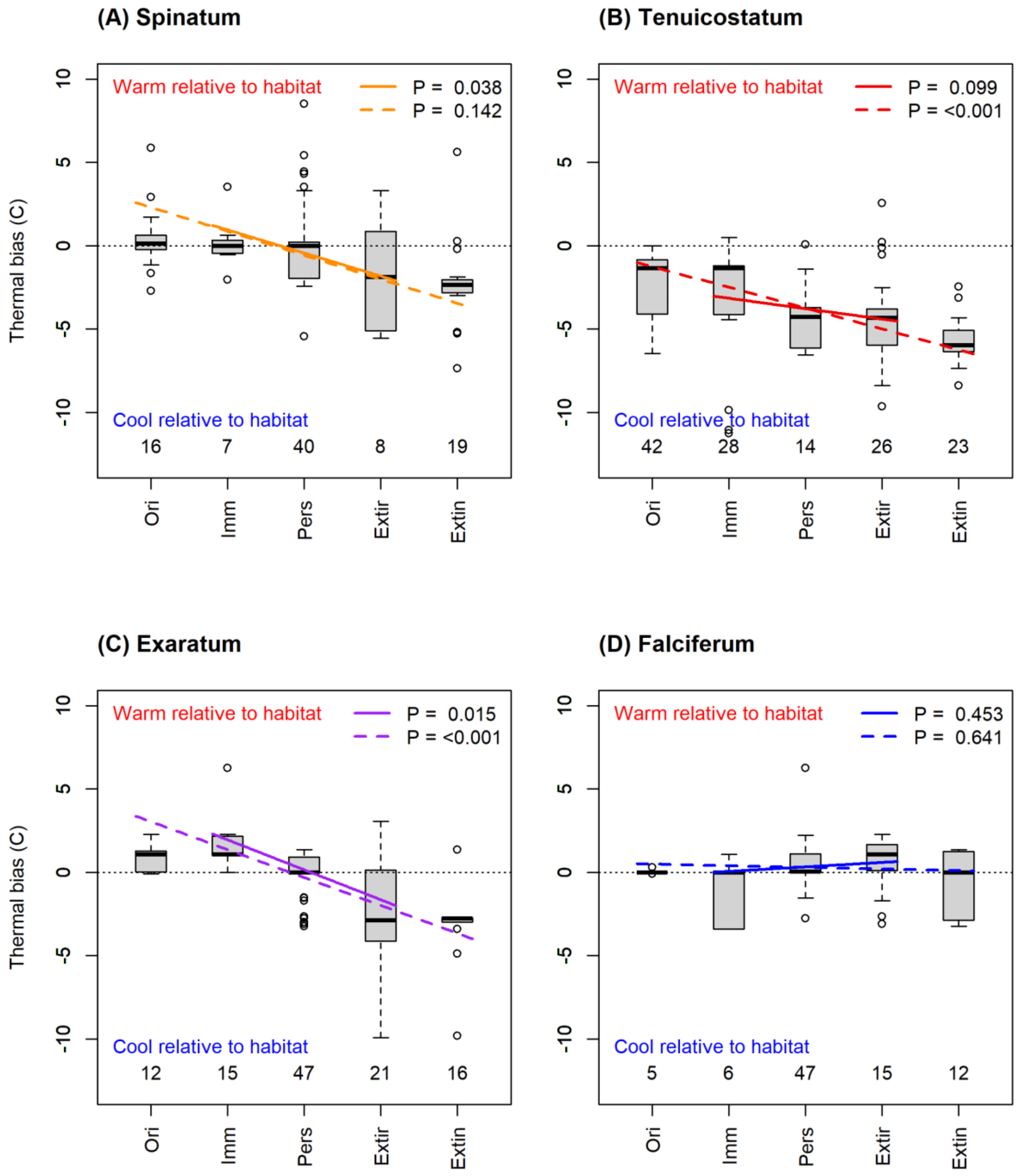

Supplementary Figure 1. Species response to climate change using three-timer species. Panels show the central (focal) ammonite zones of the three-bin window (refer to Supplementary Figure 10), including the (A) Spinatum, (B) Tenuicostatum, (C) Exaratum and (D) Falciferum zones. Each panel shows two regressions, the solid line regressions are run across immigrating, persisting, and extirpated species only, the dashed line regressions are run across all five occupancy response levels. Regions nested within zone regression models. Values along x-axis are the numbers of species per occupancy response level for each time zone. Source data are provided as a Source Data file.

(A)

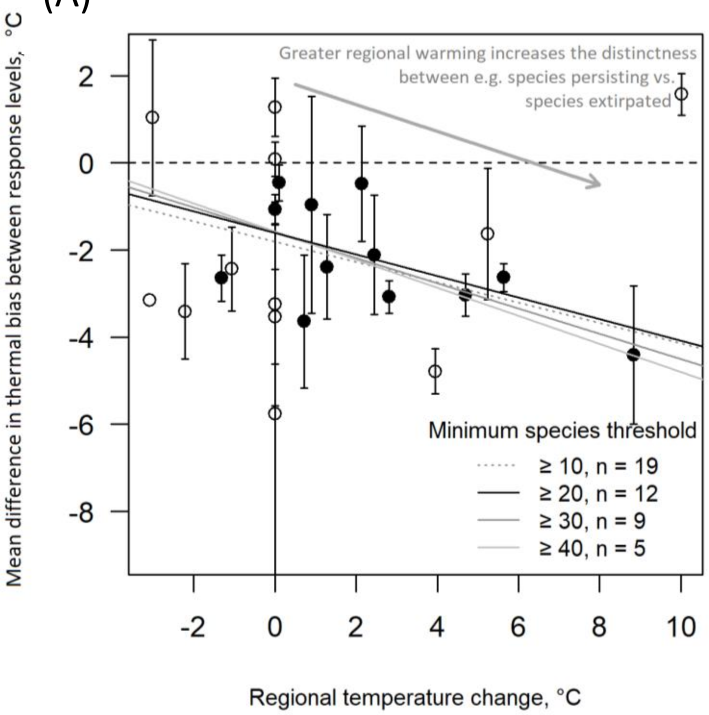

(B)

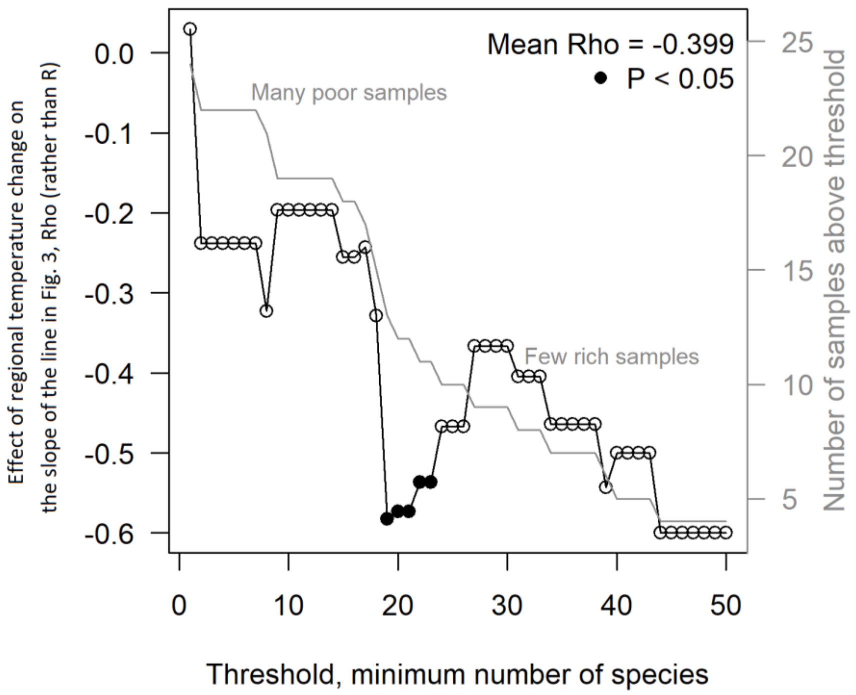

Supplementary Figure 2. Exploring how the difference in thermal bias among occupancy responses changes significance with number and quality of spatio-temporal observations. (A) Same as Fig. 3 but different lines show the relationship's dependence on the number of species in each observation and the number of spatio-temporal observations. Mean differences (y-axis) calculated from observations of more species (see legend) are more likely to support a relationship between thermal bias and the response gradient. However, few observations (n values in legend) meet these higher

thresholds of species number. Filled circles are those with at least 20 species, their regression shown by the solid black line, R = -0.25, 95% confidence intervals or CIs = -0.49—-0.01, P = 0.04, while the slopes of the other lines were insignificant (next closest was threshold = 10 with R = -0.23, P = 0.06). Direct exploration of the effect of varying the minimum number of species threshold on the relationship is shown in (Supplementary Fig. 2). All other details as in Fig. 3. (B) Direct exploration of the effect of the species number threshold on the relationship shown in Fig. 3 (left y-axis here) and number of remaining observations (right y-axis, grey), showing a compromise between quantity and quality of observations. Alongside Fig. 3, this suggested the minimum number of species required in a region and ammonite zone for the relationship between thermal bias and occupancy response to be detectable to be around 20. Source data are provided as a Source Data file.

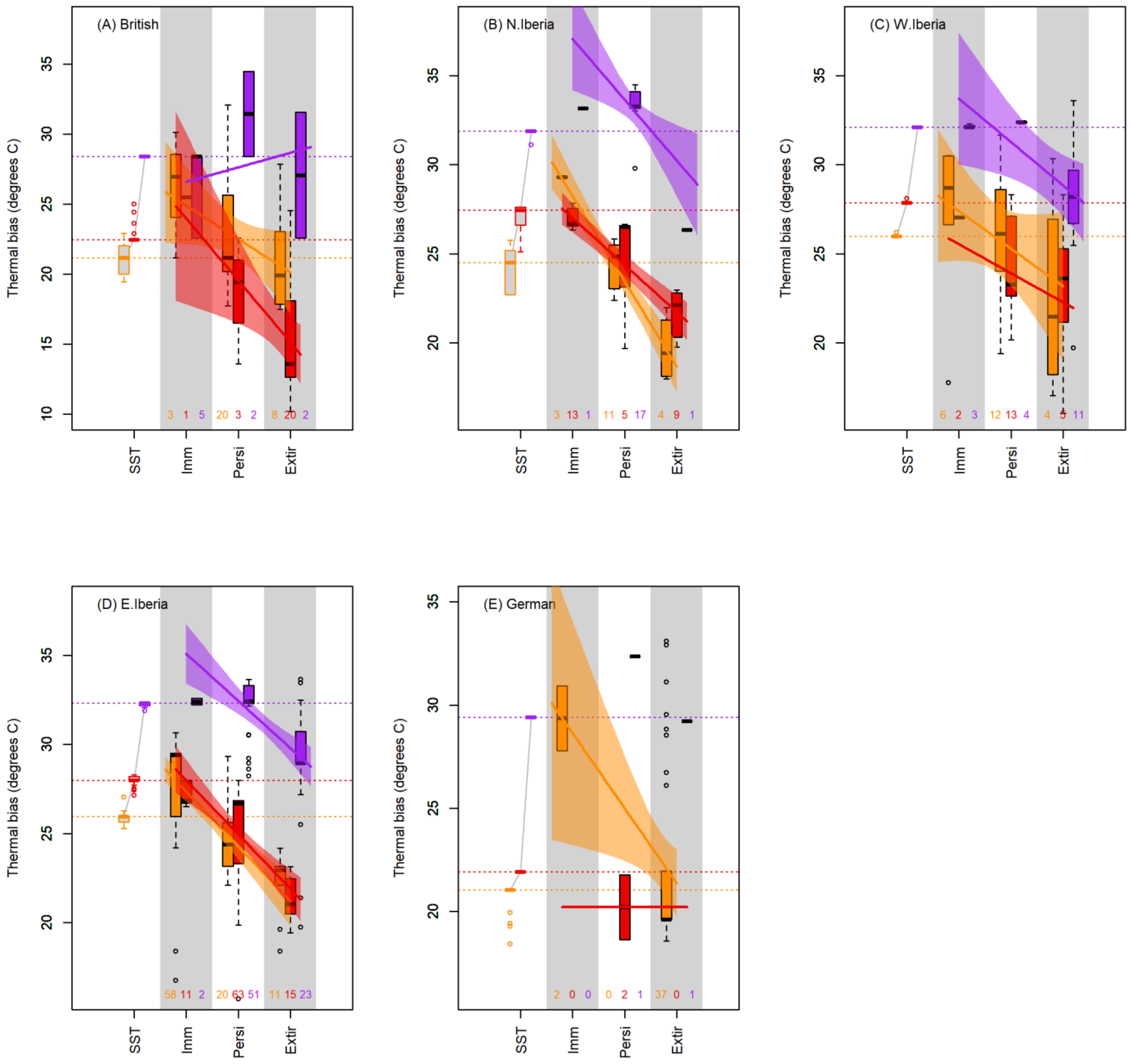

Supplementary Figure 3. Regional consistency of the correlation between species' thermal bias and whether a species is added, kept or removed from an assemblage during warming intervals. Regions cover (A) British basins, (B) north of, (C) west of, and (D) east of Iberia, and (E) Germanic basins. The left-most three boxplots per panel show environmental warming at occurrences (SST = sea surface temperatures; caution that occurrence-based temperature changes are influenced by changes in sampling paleolatitude) over the time zones, coloured and ordered from Spinatum

(orange), Tenuicostatum (red), and Exaratum (purple). Species occupancy response abbreviations are ‘Imm’ for immigrations, ‘Persi’ for persisting, and ‘Extir’ for extirpations. Regression 95% confidence intervals plotted only if $P < 0.2$. Values along x-axis are the numbers of species for each time zone and species occupancy response level, with originating and immigrating species pooled, and species going extinct or extirpated pooled. Source data are provided as a Source Data file.

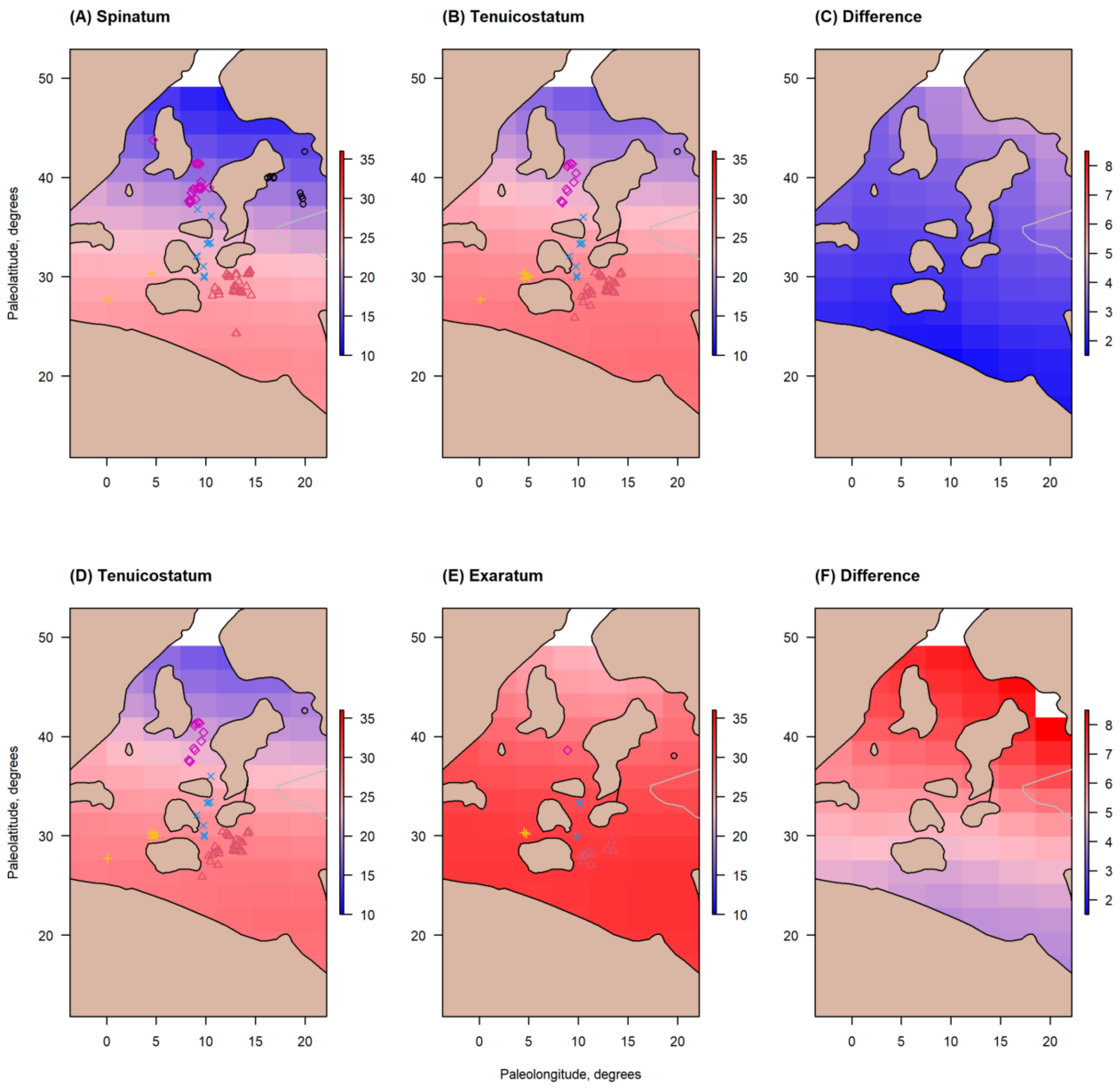

Supplementary Fig. 4. Comparing geographical patterns of SST over the two warming phases. Panels A, B, D and E use the same colour scale, highlighting how cooler habitats disappeared from the northwestern Tethys over time. Panels C and F use the same colour scale as each other, highlighting how mean warming may have been greatest in northern regions (note that panel C does not capture rapid warming over the stage boundary at this temporal resolution). These (sub)zones cover the range of temperature values of the main CO_2 scenario, from Spinatum at 400 ppm to Exaratum at 1000 ppm (see Table 2 and global maps, Supplementary Fig. 8, for additional scenarios). For comparison with Fig. 1, these show the Toarcian paleogeography (i.e. maximum sea-level coastlines for the study interval). The coverage of occurrences per (sub)zone is shown (A, B, D, E only) using the same colouration as Fig. 1. Source Data are provided as a Source Data file.

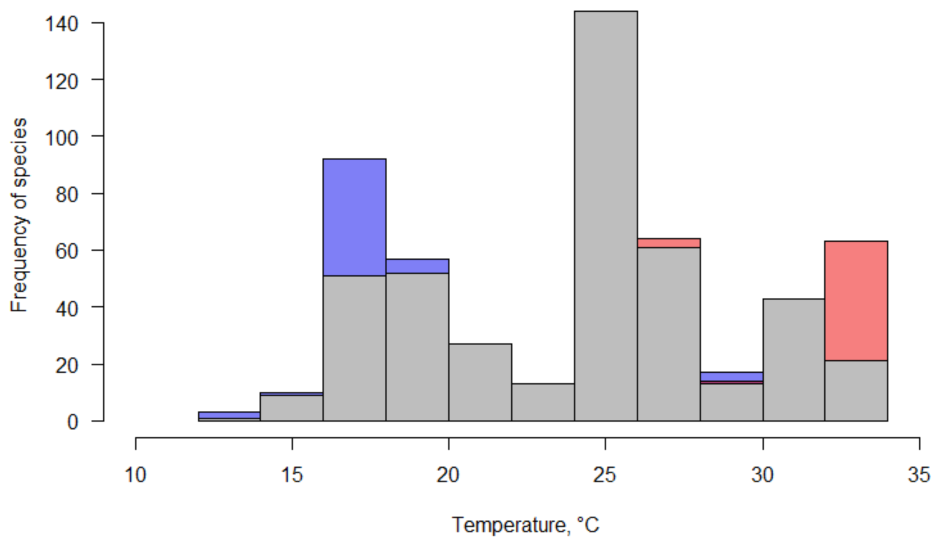

Supplementary Fig. 5. Histogram of this study's species' temperature medians (grey), and 5% and 95% percentiles (blue and pink, respectively) over our complete dataset. Many species have distributions centred around 25°C with recorded upper limits around 33.5°C and lower limits around 16.5°C. Source data are provided as a Source Data file.

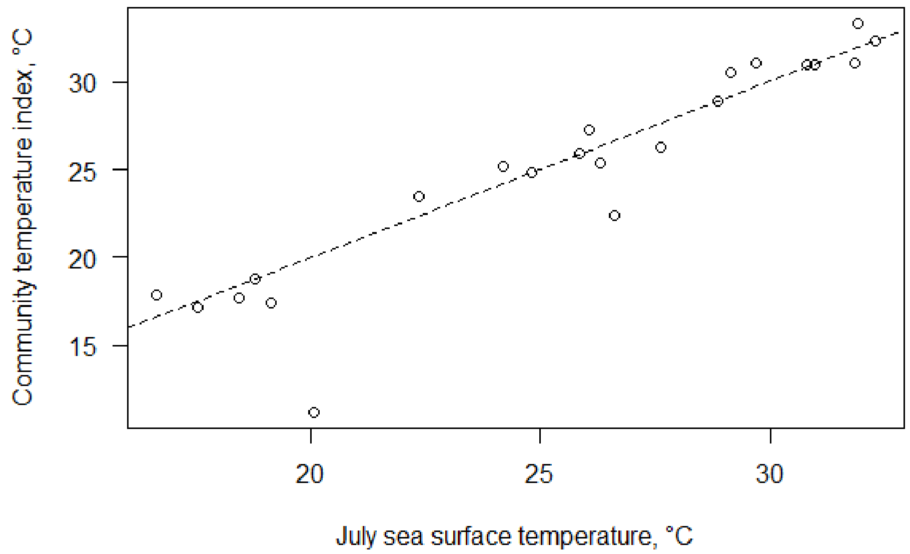

Supplementary Fig. 6. Community temperature index (i.e. the median over the species temperature indices, STI, of all species in an assemblage) for our data in relation to seawater temperatures. Each dot represents a regional assemblage observation in an ammonite (sub)zone. The dashed line represents the 1:1 correlation as an expectation of CTI (i.e. communities on average in thermal equilibrium; with a slope R = 1). The observed regression slope was close, R = 0.92 ± 0.09, $P < 0.0001$, $R^2_{adj} = 0.81$. Seawater temperatures are the regional mean for the (sub)zone over the fossil occurrences. Source data are provided as a Source Data file.

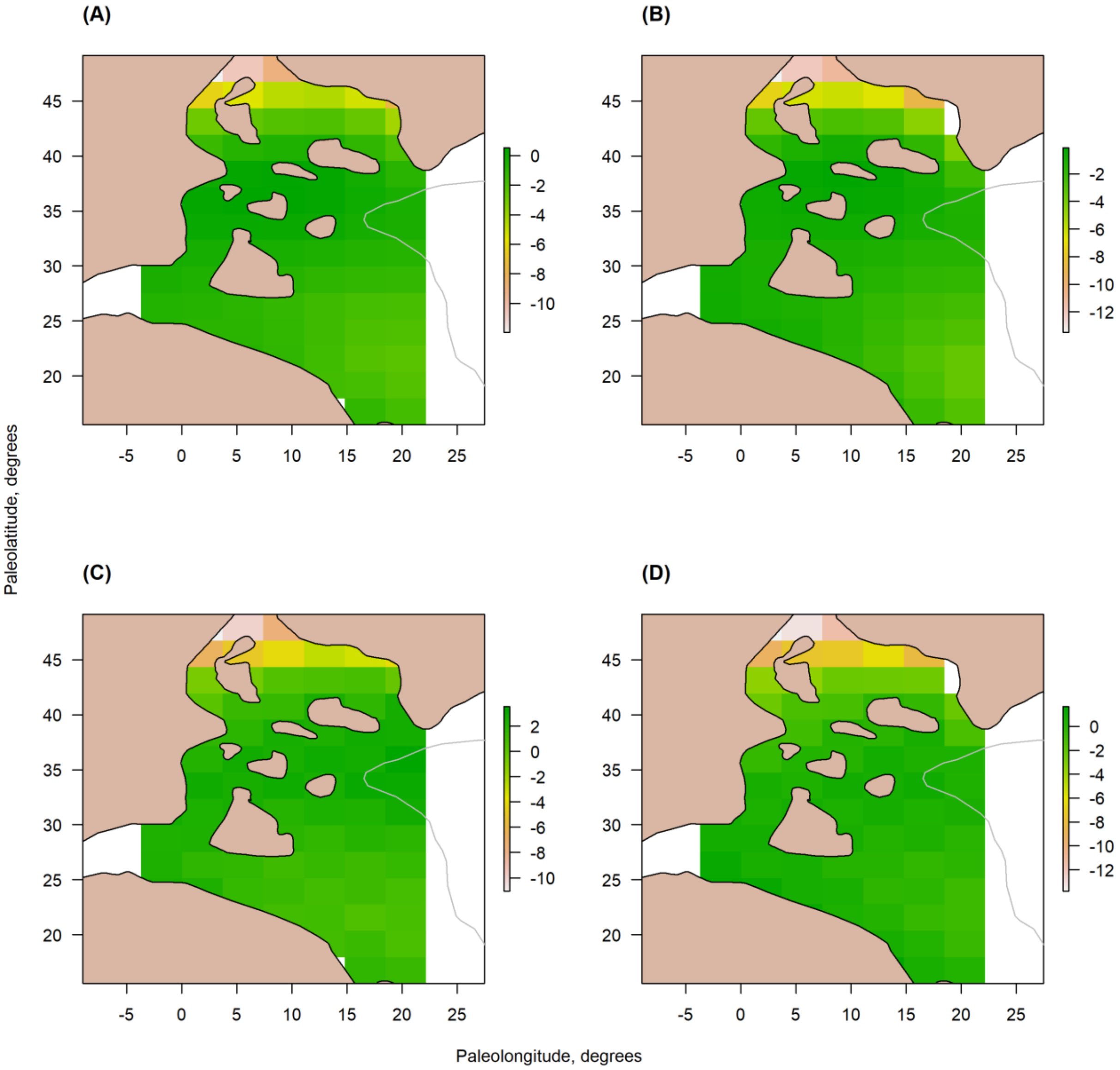

Supplementary Figure 7. Temperature differences between HadCM3 – CLIMBER-X SST outputs for different paleogeographical model and CO_2 scenarios. Orange and grey colours in the colour key (degrees C) highlight regions where the CLIMBER-X outputs were warmer, while green colours show closer agreement. (A) 185 Ma paleogeography and low CO_2, (B) 185 Ma paleogeography and high CO_2, (C) 180 Ma paleogeography and low CO_2, (D) 180 Ma paleogeography and high CO_2. Coastlines from maximum sea level transgression during Pliensbachian (185Ma). Source data are provided as a Source Data file.

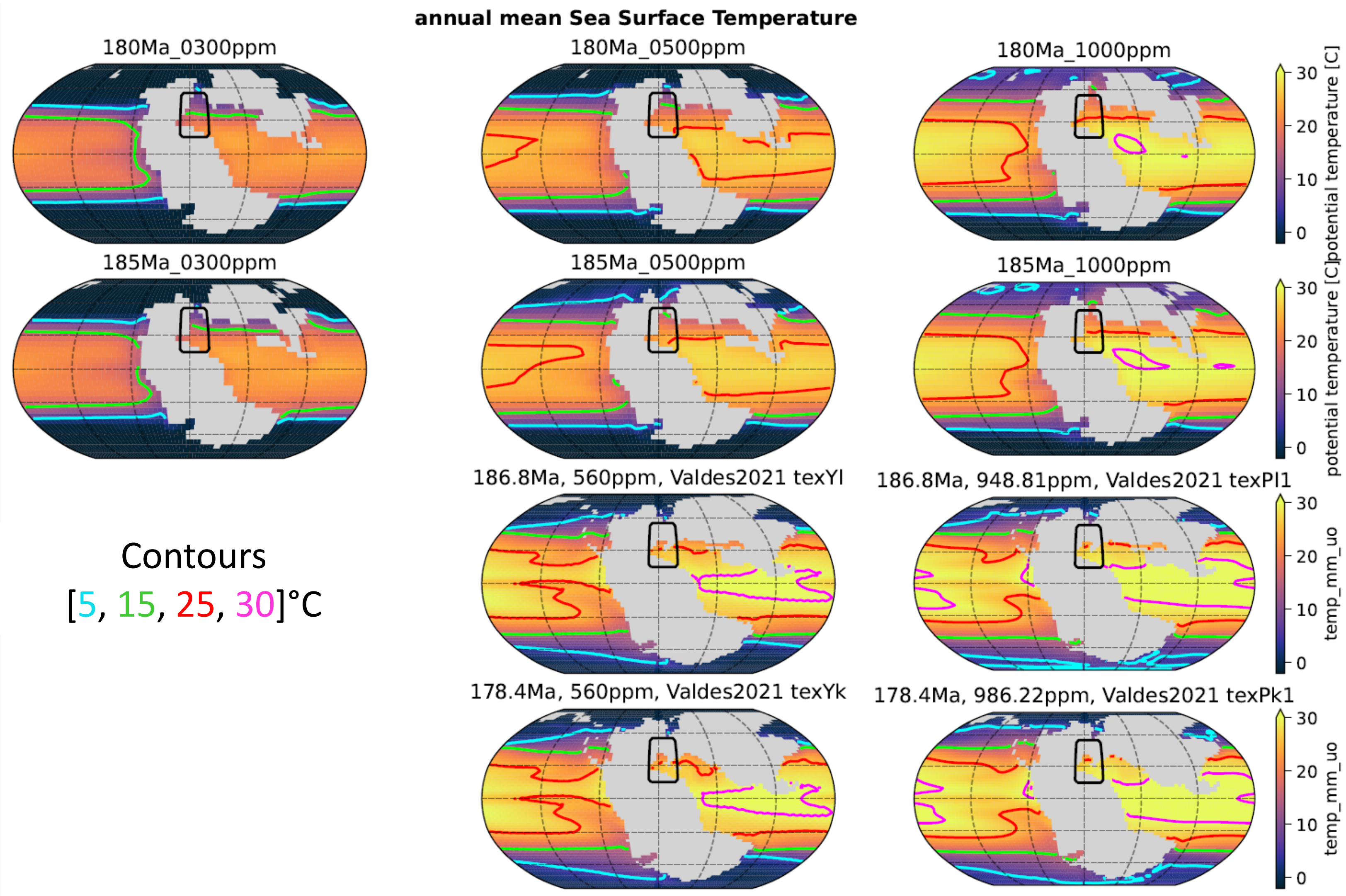

Supplementary Figure 8. Global maps of the outputs from CLIMBER-X and HadCM3 models across CO_2 levels and paleogeographical scenarios (185 or 186.8Ma being Pliensbachian and 180 or 178.4Ma being Toarcian). A box highlights the focal region on all maps.

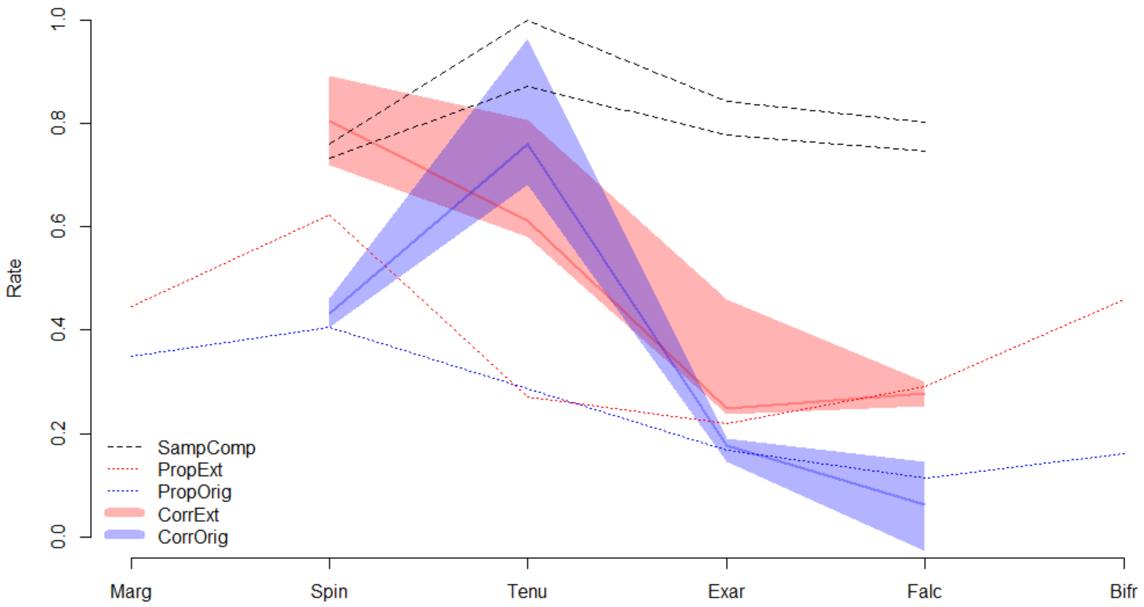

Supplementary Figure 9. Large difference between raw and corrected rates of originations and extinctions. Red lines are extinction rates, blue are origination rates. Uncorrected versions (taking data at face value) are dotted and corrected versions calculated by the per capita [26], three-timer [3], three-timer corrected for sampling completeness [3], and gap-filler rate [27] approaches, with the median over these four rates plotted (bold lines) and the range (polygon). 'SampComp' are two sampling completeness rates [3,26].

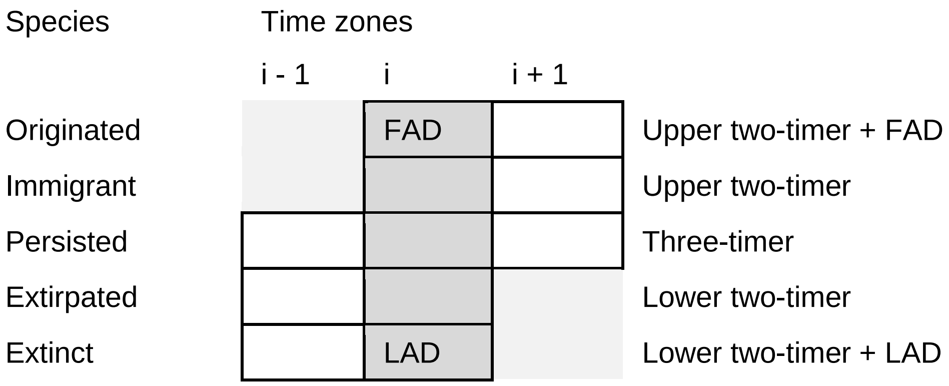

Supplementary Figure 10. Alternative, three-timer approach for designation of region-specific species responses observed over a focal bin. Focal bin highlighted in dark grey. Contrast with the main approach shown in Fig. 5. FAD = First Appearance Date. LAD = Last Appearance Date.

Supplementary Tables

Supplementary Table 1. Species occupancy response is dependent on species thermal bias, despite completely omitting species with extinction or origination responses. Number of observations n = 317, bivalves n = 197, rhynchonelliform brachiopods n = 120. $R^2_{marginal}$ = 0.17, $R^2_{conditional}$ = 0.67. Significance testing was two-tailed

	Value	S.E.	*t*	*p*
(Intercept)	3.01	0.12	25.56	6.73e-77
Thermal bias °C	-0.06	0.01	-6.13	0.002
Clade_Rhynchonelliformea	-0.12	0.07	-1.68	0.094
Regional temperature change °C	-0.02	0.03	-0.58	0.579
Thermal_bias:Clade_Rhynchonelliformea	-0.02	0.02	-1.15	0.302
Clade_Rhynchonelliformea:Regional_temp. change	0.06	0.02	3.05	0.003

Supplementary Table 2. Species occupancy response is dependent on species thermal bias, without pooling extinct species together with extirpated species, and originating species together with immigrating species. Number of observations n = 431, bivalves n = 275, rhynchonelliform brachiopods n = 156. $R^2_{marginal}$ = 0.32, $R^2_{conditional}$ = 0.54. Significance testing was two-tailed.

	Value	S.E.	*t*	*p*
(Intercept)	2.92	0.17	17.56	1.58e-51
Thermal bias °C	-0.14	0.01	-10.04	1.46e-08
Clade_Rhynchonelliformea	0.01	0.12	0.12	0.901
Regional temperature change °C	-0.06	0.04	-1.53	0.164
Thermal_bias:Clade_Rhynchonelliformea	-0.05	0.03	-1.74	0.099
Clade_Rhynchonelliformea:Regional_temp. change	0.10	0.03	3.11	0.002

Supplementary Table 3. Rerunning analyses with different paleogeographic reconstructions and alternative CO_2 scenarios for ammonite zones to assess their effect on how variables like thermal bias relate to species occupancy responses. For comparison against Table 1, which applies the main CO_2 scenario and a Toarcian base map. Significance testing was two-tailed.

(A) Pliensbachian main CO_2 scenario	Value	S.E.	*t*	*p*
(Intercept)	2.98	0.11	26.21	1.09e-88
Thermal bias °C	-0.11	0.01	-9.83	1.98e-08
Clade_Rhynchonelliformea	-0.05	0.07	-0.71	0.479
Regional temperature change °C	-0.05	0.04	-1.32	0.224
Thermal_bias:Clade_Rhynchonelliformea	-0.04	0.02	-1.93	0.070
Regional_temp. change:Clade_Rhynchonelliformea	0.08	0.02	3.30	0.001
(B) Toarcian alternative CO_2 scenario				
(Intercept)	2.98	0.11	26.01	7.07e-88
Thermal bias °C	-0.07	0.01	-10.81	4.85e-09
Clade_Rhynchonelliformea	-0.18	0.08	-2.29	0.023
Regional temperature change °C	-0.03	0.02	-1.66	0.135
Thermal_bias:Clade_Rhynchonelliformea	-0.02	0.01	-2.16	0.046
Regional_temp. change:Clade_Rhynchonelliformea	0.06	0.01	4.00	7.46e-05
(C) Pliensbachian alternative CO_2 scenario				
(Intercept)	3.02	0.12	26.30	4.64e-89
Thermal bias °C	-0.07	0.01	-10.05	1.43e-08
Clade_Rhynchonelliformea	-0.25	0.08	-3.13	0.002
Regional temperature change °C	-0.04	0.02	-2.08	0.071
Thermal_bias:Clade_Rhynchonelliformea	-0.03	0.01	-2.30	0.034
Regional_temp. change:Clade_Rhynchonelliformea	0.07	0.01	4.68	4.01e-06

Supplementary Table 4. Evaluating the impact of facies changes on results. (A) Analysis over the two warming and transition phases including facies changes. (B) Analysis over the two warming and transition phases including facies changes but excluding east of Iberia for the two warming phases. Significance testing was two-tailed.

**(A)**		Value	S.E.	*t*	*p*
	(Intercept)	3.24	0.24	13.52	3.24e-35
	Thermal bias °C	-0.15	0.01	-13.40	8.40e-11
	Regional temperature change °C	-0.08	0.05	-1.54	0.16
	Carbonate increase	0.29	0.57	0.52	0.62
	Deep increase	-0.75	0.22	-3.44	0.007
**(B)**					
	(Intercept)	3.28	0.28	11.55	1.24e-25
	Thermal bias °C	-0.13	0.01	-9.48	0.0007
	Regional temperature change °C	-0.06	0.06	-1.00	0.35
	Carbonate increase	0.07	0.50	0.14	0.89
	Deep increase	-0.71	0.18	-3.96	0.005

Supplementary Table 5. The predominance of occurrence substrate type or water depth category at each region over time. NA is where regions were represented by <10 occurrences. Grey shading when >50% occurrences were from siliciclastic substrates, or when >50% occurrences were from shallow depths. Thus, switches from shaded to unshaded, or vice versa, highlight large changes in (sampled) environment.

Zone/Subzone	Region	Proportion of all occurrences	
		Carbonate	Deep
Bifrons	British	0.00	0.02
Falciferum	British	0.03	0.94
Exaratum	British	NA	NA
Tenuicostatum	British	0.01	0.94
Spinatum	British	0.22	0.28
Margaritatus	British	0.06	0.57
Bifrons	N.Iberia	1.00	0.00
Falciferum	N.Iberia	0.84	0.00
Exaratum	N.Iberia	0.61	0.00
Tenuicostatum	N.Iberia	0.99	0.30
Spinatum	N.Iberia	0.65	0.33
Margaritatus	N.Iberia	0.83	0.00
Bifrons	W.Iberia	1.00	1.00
Falciferum	W.Iberia	1.00	1.00
Exaratum	W.Iberia	1.00	0.94
Tenuicostatum	W.Iberia	0.98	0.96
Spinatum	W.Iberia	1.00	0.18
Margaritatus	W.Iberia	1.00	0.00
Bifrons	E.Iberia	0.81	0.75
Falciferum	E.Iberia	0.85	0.85
Exaratum	E.Iberia	0.78	0.97
Tenuicostatum	E.Iberia	0.93	0.90
Spinatum	E.Iberia	0.98	0.46
Margaritatus	E.Iberia	1.00	0.57
Bifrons	Germanic	NA	NA
Falciferum	Germanic	NA	NA
Exaratum	Germanic	NA	NA
Tenuicostatum	Germanic	NA	NA
Spinatum	Germanic	0.00	0.97
Margaritatus	Germanic	0.07	0.86

Supplementary Table 6. Regional-scale sampling probability metrics through the named temporal bin. (A) range-based sampling completeness [2], (B) three-timer sampling completeness [3], (C) three-timer sampling completeness of carbonate habitats. Grey cells highlight poorer completeness values <0.66.

	Region	Spinatum	Tenuicostatum	Exaratum	Falciferum
(A)	Germanic	NA	NA	1.00	0.00
	E.Iberia	0.50	1.00	0.92	0.80
	W.Iberia	1.00	1.00	0.75	0.33
	N.Iberia	0.86	1.00	0.67	1.00
	British	0.81	0.63	0.10	0.50
(B)	German	NA	NA	1.00	0.00
	E.Iberia	0.50	1.00	0.91	0.80
	W.Iberia	1.00	1.00	1.00	0.50
	N.Iberia	0.86	1.00	0.67	1.00
	British	0.89	NA	0.25	0.67
(C)	German	NA	NA	NA	0.00
	E.Iberia	0.50	1.00	0.86	0.67
	W.Iberia	1.00	1.00	1.00	0.50
	N.Iberia	0.86	1.00	0.60	1.00
	British	NA	NA	NA	NA

Supplementary Table 7. Exploring how the thermal suitability of the cooler and warmer-adapted members of the assemblage affected the occupancy response of an assemblage (%) during warming and transition phases. Here, the 0.25, 0.5, and 0.75 quantiles of species thermal bias were used to represent the thermal bias of the relatively cooler adapted species within an assemblage, the central tendency over all species in the assemblage (median), or the warmer adapted members of the assemblage, respectively. Rho quantifies the correlation between thermal bias and % of regional assemblage. Significance testing was two-tailed.

Quantile	Rho	P		Assemblage response
0.25	-0.011	0.502		Extinct
0.25	-0.026	0.118		Extirpate
0.25	0.071	0.058	.	Persist
0.25	-0.025	0.084	.	Immigrate
0.25	-0.013	0.004	**	Originate
0.25	-0.040	0.196		Turnover
0.5	-0.003	0.853		Extinct
0.5	-0.022	0.216		Extirpate
0.5	0.024	0.465		Persist
0.5	-0.026	0.080	.	Immigrate
0.5	-0.013	0.002	**	Originate
0.5	-0.041	0.215		Turnover
0.75	-0.011	0.529		Extinct
0.75	-0.022	0.191		Extirpate
0.75	0.049	0.213		Persist
0.75	-0.017	0.263		Immigrate
0.75	-0.011	0.007	**	Originate
0.75	-0.028	0.285		Turnover

Supplementary Table 8. Regional salinity medians (ppt) at different CO_2 levels and paleogeographical assumptions. CO_2 levels: low = 560 ppm, high = 950 ppm. Paleogeography: Pli = 185 Ma maps, Toa = 180 Ma maps. The final column is the difference between regional maximum and minimum median salinities in ppt.

	Germanic	E. Iberia	W. Iberia	N. Iberia	British	Max minus min
ToaLow	34.25	35.11	34.57	34.31	34.15	0.96
ToaHigh	34.61	35.65	35.15	34.80	34.47	1.18
PliLow	33.32	33.97	33.93	33.49	33.36	0.65
PliHigh	33.71	34.55	34.42	34.10	33.92	0.84

Supplementary Table 9. Comparing modelled sea surface temperatures between CLIMBER-X and HadCM3 models. Spearman's Rho, Root Mean Squared Error (RMSE) is in degrees C, NRMSE = normalised RMSE. Green shows the best agreement by NRMSE.

Paleogeography	CO_2 scenario	CLIMBER-X	HadCM3	Rho	RMSE	NRMSE
180 Ma	High	clim180.750KS	clim178.950KS	0.81	3.06	0.17
180 Ma	High	clim180.1000KS	clim178.950KS	0.81	4.02	0.22
180 Ma	High	clim180.1250KS	clim178.950KS	0.81	4.81	0.27
180 Ma	High	clim180.1500KS	clim178.950KS	0.81	5.45	0.30
185 Ma	High	clim185.750KS	clim187.950KS	0.78	3.67	0.19
185 Ma	High	clim185.1000KS	clim187.950KS	0.78	4.81	0.25
185 Ma	High	clim185.1250KS	clim187.950KS	0.78	5.67	0.30
185 Ma	High	clim185.1500KS	clim187.950KS	0.77	6.35	0.33
180 Ma	Low	clim180.300KS	clim178.560KS	0.96	4.38	0.18
180 Ma	Low	clim180.400KS	clim178.560KS	0.96	2.75	0.11
180 Ma	Low	clim180.500KS	clim178.560KS	0.96	3.67	0.15
180 Ma	Low	clim180.750KS	clim178.560KS	0.94	8.00	0.33
185 Ma	Low	clim185.300KS	clim187.560KS	0.95	4.23	0.18
185 Ma	Low	clim185.400KS	clim187.560KS	0.95	3.04	0.13
185 Ma	Low	clim185.500KS	clim187.560KS	0.95	4.75	0.20
185 Ma	Low	clim185.750KS	clim187.560KS	0.90	7.73	0.32

Supplementary Table 10. Comparing model results from contrasting scenarios of paleogeography and CO_2 levels. For example, '185.Low' shows the results for a Pliensbachian paleogeography and lowest CO_2 levels (300 ppm in CLIMBER-X model, 560ppm in HadCM3 model).

CLIMBER-X scenario	HadCM3 scenario	CLIMBER-X	HadCM3	Rho	RMSE	NRMSE
185.Low	180.High	clim180.1500KS	clim187.560KS	0.95	10.46	0.44
180.Low	185.High	clim185.1500KS	clim178.560KS	0.92	10.79	0.45
185.High	180.Low	clim180.300KS	clim187.950KS	0.80	8.21	0.43
180.High	185.Low	clim185.300KS	clim178.950KS	0.77	8.66	0.48
