# Supplementary material for: Marine species and assemblage change foreshadowed by their thermal bias over Early Jurassic warming
Source: Nat Commun. 2025 Feb 5;16:1370. doi: 10.1038/s41467-025-56589-0 (PMC11799210; doi:10.1038/s41467-025-56589-0)
Supplement: Supplementary file 3 — Description of Additional Supplementary Files [file 41467_2025_56589_MOESM3_ESM.pdf]

Description of Additional Supplementary Files to “Marine species and assemblage change foreshadowed by their thermal bias over Early Jurassic warming”

Carl J. Reddin et al.

This document provides legends for Supplementary Data 1 to 3 , which supplement our paper, “Marine species and assemblage change foreshadowed by their thermal bias over Early Jurassic warming”.

Supplementary Data 1. Taxonomic vetting of species names from those downloaded (identified name at least accepted at genus level). Columns are taxonomic class, identified name (as initially entered into PBDB; downloaded), accepted name (based on PBDB accepted taxonomic names and opinions; downloaded), final vetted species name (used to determine species regional responses in our analysis; not downloaded); rows are the unique combinations of these entries e.g. a single vetted species name may have multiple identified names. Note that all species are listed, not only the two-timers.

Supplementary Data 2. Stratigraphic binning of occurrences to the final ‘ammonite zone’ from the information downloaded from the PBDB. Columns used: ‘early_interval’, ‘zone’, and, if necessary, ‘stratcomments’ (in order of decreasing priority). For example, if ‘early_interval’ was set to ‘Late Pliensbachian’, first the ‘zone’ column was checked, and if insufficient, the ‘stratcomments’ column was checked. Only unique combinations of information are shown rather than every single occurrence. Occurrences where a final ‘ammonite zone’ could not be designated were not used for the main analysis so are not shown here. Cells and details are top-left aligned. Entries in “stratcomments” are taken as they appear in the PBDB.

Supplementary Data 3. Full references of data sources used in this study ranked by the number of utilised occurrences (no. occ. utilised). Data were accessed by the PaleoBioDB (see Methods).
