# Supplementary material for: Marine species and assemblage change foreshadowed by their thermal bias over Early Jurassic warming
Source: Nat Commun. 2025 Feb 5;16:1370. doi: 10.1038/s41467-025-56589-0 (PMC11799210; doi:10.1038/s41467-025-56589-0)
Supplement: Supplementary file 7 — Reporting Summary [file 41467_2025_56589_MOESM7_ESM.pdf]

nature portfolio

Corresponding author(s): Carl J Reddin

Last updated by author(s): Jan 10, 2025

Reporting Summary

Nature Portfolio wishes to improve the reproducibility of the work that we publish. This form provides structure for consistency and transparency in reporting. For further information on Nature Portfolio policies, see our Editorial Policies and the Editorial Policy Checklist.

Statistics

For all statistical analyses, confirm that the following items are present in the figure legend, table legend, main text, or Methods section.

n/a	Confirmed	
☐	☒	The exact sample size (*n*) for each experimental group/condition, given as a discrete number and unit of measurement
☐	☒	A statement on whether measurements were taken from distinct samples or whether the same sample was measured repeatedly
☐	☒	The statistical test(s) used AND whether they are one- or two-sided<br>*Only common tests should be described solely by name; describe more complex techniques in the Methods section.*
☐	☒	A description of all covariates tested
☐	☒	A description of any assumptions or corrections, such as tests of normality and adjustment for multiple comparisons
☐	☒	A full description of the statistical parameters including central tendency (e.g. means) or other basic estimates (e.g. regression coefficient) AND variation (e.g. standard deviation) or associated estimates of uncertainty (e.g. confidence intervals)
☐	☒	For null hypothesis testing, the test statistic (e.g. *F*, *t*, *r*) with confidence intervals, effect sizes, degrees of freedom and *P* value noted<br>*Give P values as exact values whenever suitable.*
☒	☐	For Bayesian analysis, information on the choice of priors and Markov chain Monte Carlo settings
☐	☒	For hierarchical and complex designs, identification of the appropriate level for tests and full reporting of outcomes
☐	☒	Estimates of effect sizes (e.g. Cohen's *d*, Pearson's *r*), indicating how they were calculated

Our web collection on statistics for biologists contains articles on many of the points above.

Software and code

Policy information about availability of computer code

Data collection	Custom R scripts and versions are publicly available at Zenodo repository, 10.5281/zenodo.14626268
Data analysis	Custom R scripts and versions are publicly available at Zenodo repository, 10.5281/zenodo.14626268

For manuscripts utilizing custom algorithms or software that are central to the research but not yet described in published literature, software must be made available to editors and reviewers. We strongly encourage code deposition in a community repository (e.g. GitHub). See the Nature Portfolio guidelines for submitting code & software for further information.

Data

Policy information about availability of data

All manuscripts must include a data availability statement. This statement should provide the following information, where applicable:

- Accession codes, unique identifiers, or web links for publicly available datasets
- A description of any restrictions on data availability
- For clinical datasets or third party data, please ensure that the statement adheres to our policy

The R code used for the main analysis, the raw data, the processed fossil occurrence data, and associated climate data, and instructions are publicly available from Zenodo repository, 10.5281/zenodo.14626268

Research involving human participants, their data, or biological material

Policy information about studies with human participants or human data. See also policy information about sex, gender (identity/presentation), and sexual orientation and race, ethnicity and racism.

Reporting on sex and gender	N/A
Reporting on race, ethnicity, or other socially relevant groupings	N/A
Population characteristics	N/A
Recruitment	N/A
Ethics oversight	N/A

Note that full information on the approval of the study protocol must also be provided in the manuscript.

Field-specific reporting

Please select the one below that is the best fit for your research. If you are not sure, read the appropriate sections before making your selection.

☐ Life sciences ☐ Behavioural & social sciences ☒ Ecological, evolutionary & environmental sciences

For a reference copy of the document with all sections, see nature.com/documents/nr-reporting-summary-flat.pdf

Ecological, evolutionary & environmental sciences study design

All studies must disclose on these points even when the disclosure is negative.

Study description	Here, we examined the effects of regional warming on marine benthic species occupancy and assemblage composition over one-million-year time steps during the Early Jurassic. Thermal bias, the difference between modelled regional temperatures and species' long-term thermal optima, predicted species responses to warming in an escalatory order.
Research sample	The fossil occurrence data are previously published and publicly available on the PaleoBioDB (https://paleobiodb.org/). The occurrences are species-level presence/absence at a geographical location and time bin, the ammonite zones. Occurrences are pooled to represent presence/absence of regional clusters per time bin, which may resemble species ecological populations (see 'Spatial clusters' in methods; though this is not necessary for the importance of our results). The species used are all 'two-timers', which are sampled in the same region over at least two consecutive time bins, representing the better sampled species.
Sampling strategy	The important sample size was the regional cluster, which was calculated by hierarchical clustering of all occurrence geographical coordinates using Euclidean distances. Practical requirements for regional clusters included (1) being sampled in different time steps, ideally throughout, and (2) having sufficient occurrences.
Data collection	Fossil occurrence data were previously collected by other scientists at rock outcrops worldwide and published in the papers listed in Supplementary Table 13. These data were then entered into the publicly available on the PaleoBioDB (https://paleobiodb.org/) either by us or by other data enterers (the majority of the data was entered by others).
Timing and spatial scale	The temporal focus is from the Late Pliensbachian Margaritatus ammonite zone (beginning around 187 Ma) to the middle Toarcian Bifrons ammonite zone (around 180 Ma). The ammmonite zones had an approximate mean duration of 1.1 myr, forming the temporal resolution of this study. Geographical regions varied slightly in size and spatial dimensions but mean width is approximately ~2000 km.
Data exclusions	Occurrences initially had to be accepted at least at the genus level and required modern geographical coordinates to allow paleogeographical rotation. Confidently identified species that could not be accepted in the taxonomic vetting were also excluded. These data were used to calculate species FADs and LADs.<br><br>Species for analysis. In addition to the above, we focus on north-west Tethys occurrences by using a bounding box around modern Europe, excluding occurrences outside the bounding box and excluding those with coarser than ammonite zone resolution or that fell outside the focal ammonite zones. Species were all 'two-timers', which were sampled in the same region over at least two consecutive time bins, representing the better sampled species.<br><br>Spatial clusters were set to eight to maximise agreement between purely geographical considerations (Euclidean distances) with ecological considerations (clustering by Jaccard's similarity of species occurrences; see 'Spatial clusters' in methods). Practical requirements for spatial clusters included (1) being sampled in different time steps, ideally throughout, and (2) having sufficient occurrences, resulting in a further three clustering being dropped.
Reproducibility	The R-code for analyses will publicly available from Zenodo repository, 10.5281/zenodo.14626268. Our main analyses were also reanalysed under different assumptions, including the use of three-timer species rather than two-timer species (see Methods and Supplementary Fig.1), and excluding the categories of 'extinction' and 'origination', to check whether relationships were dependent

on these categories.

Randomization

Known multiple, strong systematic biases often mean, we believe, that pure randomisation is a poor means of extracting any ecological signal from the fossil record. In other words, the biases demonstratibly remain after random sampling. Instead, we take the approach of focussing on the better sampled geographical regions (clusters), times (ammonite zones), and species (two-timers), thereby excluding the noisiest parts of the data and comparing like with like. Random effects in our analyses then calculate statistical error at a wider level of representation (i.e. the given observations represent a random sample of a wider statistical population, rather than the given observations being solely of interest).

Blinding

Analytical decisions were made a priori: how clustering would progress to define regions, the use of two-timer and three-timer species, how species would be categorised, per region per ammonite zone, as extinct, extirpated, persisting, immigrating, or originating. Climate models were run by co-authors separate to the ecological analysis team. Data vetting where carried out before analyses were begun.

Did the study involve field work? ☐ Yes ☒ No

Reporting for specific materials, systems and methods

We require information from authors about some types of materials, experimental systems and methods used in many studies. Here, indicate whether each material, system or method listed is relevant to your study. If you are not sure if a list item applies to your research, read the appropriate section before selecting a response.

Materials & experimental systems

n/a	Involved in the study	
☒	☐	Antibodies
☒	☐	Eukaryotic cell lines
☐	☒	Palaeontology and archaeology
☒	☐	Animals and other organisms
☒	☐	Clinical data
☒	☐	Dual use research of concern
☒	☐	Plants

Methods

n/a	Involved in the study	
☒	☐	ChIP-seq
☒	☐	Flow cytometry
☒	☐	MRI-based neuroimaging

Palaeontology and Archaeology

Specimen provenance

N/A. We only used publicly available data from published journal articles. No permits were required as no physical specimens were used.

Specimen deposition

N/A. We only used publicly available data from published journal articles. No physical specimens were used.

Dating methods

No new dates were provided. Data were dated to ammonite zones based on information provided in the database.

☐ Tick this box to confirm that the raw and calibrated dates are available in the paper or in Supplementary Information.

Ethics oversight

No ethical approval or guidance was required since we only used publicly available data from published journal articles; no physical specimens were used.

Note that full information on the approval of the study protocol must also be provided in the manuscript.

Plants

Seed stocks

N/A

Novel plant genotypes

N/A

Authentication

N/A
